# Scientific Items

**Published:** 1881-02-20

**Authors:** 


### SCIENTIFIC ITEMS.

**Ozone**.—Ozone is a modification, or what is sometimes called an "allotropic," of oxygen. It differs remarkably in its properties from ordinary oxgen, being far more energetic in its action. It attacks many metals and other substances which are not affected by oxygen. It is on account of this intenser energy that it is a powerful bleaching and disinfecting agent. Its specific gravity is one-half greater than that of oxygen. It has also a peculiar odor, from which it gets its name.

As oxygen has been liquefied, we might expect that ozone (or, rather, the mixture ozone and oxygen, for the former cannot be obtained pure) would be also reduced to the liquid state; and this has been accomplished by Troost and Hautefeuille. They first succeeded in preparing ozone in a more concentrated form than had previously been known. They obtained gas containing 60 per cent. of ozone; and interesting results are got by compressing this gas, or even oxygen with 20 per cent. of ozone, in Cailletet's apparatus for the liquefaction of gases. Sudden compression gives rise to very brilliant luminous phenomena. Much heat is liberated, the tube flies to pieces, and the ozone is converted into ordinary oxygen. If the compression is conducted slowly, the gas becomes violet-blue, the tint growing deeper and deeper. This is evidently the color of ozone, as other experiments, which we cannot describe here, also prove. When the ozone, after being subjected to a pressure of thirty-five atmospheres, is allowed to expend suddenly, a blue mist appears, which consists of liquefied ozone. To reduce oxygen to the same condition of pressure of three hundred atmospheres is required.

**The Color of Ozone.**—A paper recently read before the French Academy of Sciences contains some interesting facts relative to the liquefaction of ozone. A reservoir containing oxygen, at a temperature of 9·4° below zero (Fah.), is charged with ozone, and pressure applied by a column of mercury acted upon by a hydraulic press. Immediately the gas begins to turn to an azure blue color, deepening the shade as the pressure increases. The liquefactionof ozone was obtained by applying a pressure to the ozonized oxygen of 75 atmospheres, while 300 atmospheres of pressure would have been required for pure oxygen. The fact was also established that ozone is an explosive gas, since unless compressed slowly and at a low temperature, it exploded with a yellow flame. Its heavenly blue color was rendered manifest not only under heavy pressure, but under all circumstances.—*Drug. Circular.*

**The Occurrence of Tails in Men.**—Prof. Virchow, in a brief article on this subject, translated in the Medical Times, refers to several cases which have been reported by recent or older writers. Dr. Ornstein, of Athens, surgeon-in-chief of the Greek army, has recently reported several instances of abnormal growth of hair in the sacral region, which Virchow designates as "sacral trichosis." Ornstein's view was that these growths were atavic in character, and were analogous to the hairy tails of inferior animals. Virchow, having met with a case of partial lumbar trichosis, investigated the matter, and came to the conclusion that two similar but distinct conditions may exist—either a simple growth of hair or a hairless prolongation from the coccyx, of a cutas neous nature. Virchow's case appeared on examination to be an unusual form of nævus pilosus, situated over the closed spina bifida of an adult woman, and evidently to be explained by the supposition of early local irritation. But, on the other hand, medical literature certainly affords a certain number of examples of true tail formation in man, this appendage appearently resulting from elongation of the vertebral column. None of these cases, however, were complicated by the abnormal growth of hair. One of Ornstein's cases showed a distinct elongation five centimeters (two inches) in length. It appeared to originate in the attachment between the first and second false vertebræ of the coccyx. The process itself was hairless, but a decided collection of hair appeared over the sacral region.

Michel has pointed out that in the human embryo a rudimentary tail is distinctly made; and the discovery of men with tails seems to lend support to Lord Monboddo's theory that all mankind originally wore them. Virchow remarks upon the frequent occurrence of a considerable quantity of hair upon the sacral region of new-born children.

One of the longest tails on record is that reported by Greve in 1878, (Virchow's Archiv, Bd. 72, p. 120). This occurred in the case of a new-born infant, was 7·5 centimeters in length, and moved about when pricked with a needle. It was removed by an operation. Virchow recently dissected this tail, and found it not to contain any bone, cartilage, or muscle; nevertheless, it was a good substitute for a tail.

The custom among certain savage nations of attaching artificial tails to the person has been regarded by some anthropologists as a reminiscence of the happier times of tailed ancestors. Virchow, however, throws some doubt on this theory.

**The Physiology of Walking.**—M. Marey, by means of an ingenious instrument, called the "odograph," has discovered some interesting facts in regard to walking. It was ascertained that the step is longer in going up hill than in going down hill. It is shorter when a burden is carried, longer with low than with high heeled boots; longer when the sole is thick and prolonged a little beyond the foot than when it is short and flexible. It appears that the heel may be advantageously removed almost entirely; but it is disadvantageous to prolong the sole beyond a certain limit, or to give it more than a certain amount of stiffness. On level ground the step becomes longer in proportion to its frequency. In going up hill the steps are longer, but less frequent.

**Strength of Insects.**—At a meeting of the Maryland Academy of Sciences recently, Dr. Theobold showed a species of beetle and gave the following figures: Weight of beetle, two grains; weight moved by it, 5½ ounces—2,640 grains, or 1,320 times the weight of the beetle. A man weighing 150 pounds, endowed with the strength of this insect, should therefore be able to move 192,000 pounds, or nearly 100 tons.—*Scientific American.*

